# Correlation between apnea–hypopnea index and Tp‐Te interval, Tp‐Te/QT, and Tp‐Te/QTc ratios in obstructive sleep apnea

**DOI:** 10.1111/anec.12809

**Published:** 2020-10-16

**Authors:** Erdem Karacop, Handan B. Karacop

**Affiliations:** ^1^ Faculty of Medicine Department of Cardiology Bezmialem Foundation University Istanbul Turkey; ^2^ Faculty of Medicine Department of Pulmonary Medicine Bezmialem Foundation University Istanbul Turkey

**Keywords:** obstructive sleep apnea (OSA), QT and Tp‐Te, QTc ratios, Tp‐Te, Tp‐Te interval

## Abstract

**Background:**

Obstructive sleep apnea (OSA) is a highly prevalent sleep disorder associated with important cardiovascular complications including ventricular arrhythmias. Tp‐Te interval, Tp‐Te/QT, and Tp‐Te/QTc ratios are repolarization indices representing ventricular arrhythmogenic potential. These parameters are associated with ventricular arrhythmias and sudden cardiac death. The aim of this study was to investigate the correlation between apnea–hypopnea index and Tp‐Te, Tp‐Te/QT, and Tp‐Te/QTc in OSA.

**Methods:**

We screened a total of 280 patients who underwent overnight polysomnography (PSG) between the years 2012–2017 at our institution. Patients were assigned into four groups based on severity of apnea–hypopnea index: 70 with apnea–hypopnea index (AHI) <5 (control group), 71 with 5 ≤ AHI < 15, 63 with 15 ≤ AHI < 30, and 76 with AHI ≥ 30. Tp‐Te, Tp‐Te/QT, and Tp‐Te/QTc were measured.

**Results:**

Compared to control group, repolarization parameters were significantly prolonged in other groups (Tp‐Te interval: 68.3 ± 6.8, 71.8 ± 6.3, 79.1 ± 5.5, and 85.1 ± 6.4 ms, *p* < .001; Tp‐Te/QT ratio: 167.5 ± 12.7, 181.7 ± 13.0, 202.2 ± 10.0 and 219.4 ± 13.5, *p* < .001; Tp‐Te/QTc ratio: 151.1 ± 16.6, 167.6 ± 16.6, 193.7 ± 14.4, and 225.5 ± 17.0, *p* < .001). There was a significant trend toward higher Tp‐Te, Tp‐Te/QT, and Tp‐Te/QTc across higher AHI categories. In a univariate regression analysis, body mass index, smoking status, Tp‐Te, and Tp‐Te/QTc were significantly associated with the severity of AHI in OSA. Tp‐Te (OR 1.629, 95% CI 1.393–1.906, *p* < .001), Tp‐Te/QTc (OR 1,333 95% CI 1.247–1.424, *p* < .001), and smoking status (OR 5.771, 95% CI 1.025–32.479, *p* = .047) were found to be significant independent predictors of severity of AHI in a multivariate analysis, after adjusting for other risk parameters.

**Conclusions:**

Our study showed that Tp‐Te, Tp‐Te/QT, and Tp‐Te/QTc were prolonged in patients with OSA. There was significant correlation between apnea–hypopnea index and these parameters.

## 1 INTRODUCTION

Obstructive sleep apnea (OSA) is characterized by repetitive, partial, or complete closure of the upper airway resulting in desaturation of blood oxygen and fragmentation of sleep (Dempsey et al., 2010). It affects approximately 9% to 38% of general adult population (Senaratna et al., 2017). Prevalence of OSA increases with age, male sex, and obesity.

Apnea–hypopnea index (AHI) has been used to evaluate the severity of OSA and treatment outcome. AHI is the combined average number of apneas and hypopneas that occur per hour of sleep. It is categorized into mild (5–15 events/hr), moderate (15–30 events/hr), and severe (>30 events/hr) (Berry et al., 2012).

The coexistence of OSA and cardiovascular disease can be explained by several common risk factors including sedentary life, obesity, and increasing age. OSA is strikingly high among patients with heart failure (Bitter et al., 2009; Herrscher et al., 2011; Oldenburg et al., 2016), established hypertension (Haentjens et al., 2007; Montesi et al., 2012; Pedrosa et al., 2013), and arrhythmias (Gami et al., 2013; Holmqvist et al., 2015; Ryan et al., 2005). Cardiac arrhythmias including second‐degree atrioventricular block, sinus arrest, atrial and ventricular premature extrasystoles, and nonsustained ventricular tachycardia have been reported up to 50% in patients with OSA ( Guilleminault et al., 1983; Somers et al., 2008). Guilleminault et al. postulated ventricular arrhythmias predominantly premature ventricular contractions are significantly higher in OSA (66% versus 0%–12%) (Guilleminault et al., 1983; Hoffstein & Mateika, 1994). In concordance with apneic episodes, arrhythmias most commonly occur during sleep (Harbison et al., 2000). The prevalence of ventricular arrhythmias correlates with the severity of hypoxemia and AHI (Selim et al., 2016). A study carried out by Gami et al. found an association between OSA and sudden cardiac death (SCD) for the first time in 2005 (Gami et al., 2005). OSA had a peak in SCD from cardiac causes during sleeping hours (Gami et al., 2005). Several pathophysiological consequences of OSA including hypoxemia, bradyarrhythmia, sympathetic activation induced by apneic events, intrathoracic pressure changes, recurrent arousals, and atrial stretch may provoke cardiac arrhythmias (May et al., 2017; Sicouri & Antzelevitch, 1991).

There is mounting evidence pointing that alterations in ventricular repolarization cause arrhythmias and SCD due to increased heterogeneity in ventricular recovery time (Dempsey et al., 2010; May et al., 2017). Recently, three electrocardiograph‐derived indicators of ventricular repolarization abnormalities have been proposed: Tp‐Te interval, Tp‐Te/QT, and Tp‐Te/QTc ratios. Tp‐Te is the interval between the peak and the end of T wave and reflects the transmural dispersion of repolarization. Yamaguchi et al. reported patients with prolonged Tp‐Te were prone to life‐threatening ventricular arrhythmias (Yamaguchi et al., 2003). Tp‐Te is a gradient of action potential duration from endo‐ to epicardial cells; hence, it is a measure of cardiac transmural dispersion of repolarization. Prolongation of this parameter causes increased susceptibility for early afterdepolarizations. Consequently, it is associated with life‐threatening arrhythmias and SCD (Yamaguchi et al., 2003). Tp‐Te/QT and Tp‐Te/QTc are novel markers of global dispersion. Disproportional prolongation of these markers relative to QT interval plays an important role in arrhythmogenesis (Gupta et al., 2008). Nonetheless, Tp‐Te is influenced by heart rate and body weight. Therefore, Tp‐Te/QT has been proposed to be a better marker of ventricular repolarization. Kilicaslan et al. reported these parameters increased in patients with OSA undergoing overnight polysomnography (PSG) (Kilicaslan et al., 2012).

In the present study, we tried to expand the knowledge about the arrhythmogenic potential of OSA. The aim was to assess the correlation between the severity of AHI and Tp‐Te, Tp‐Te/QT, and Tp‐Te/QTc in OSA.

## 2 MATERIAL AND METHODS

### 2.1 Study population

**Figure 1:**
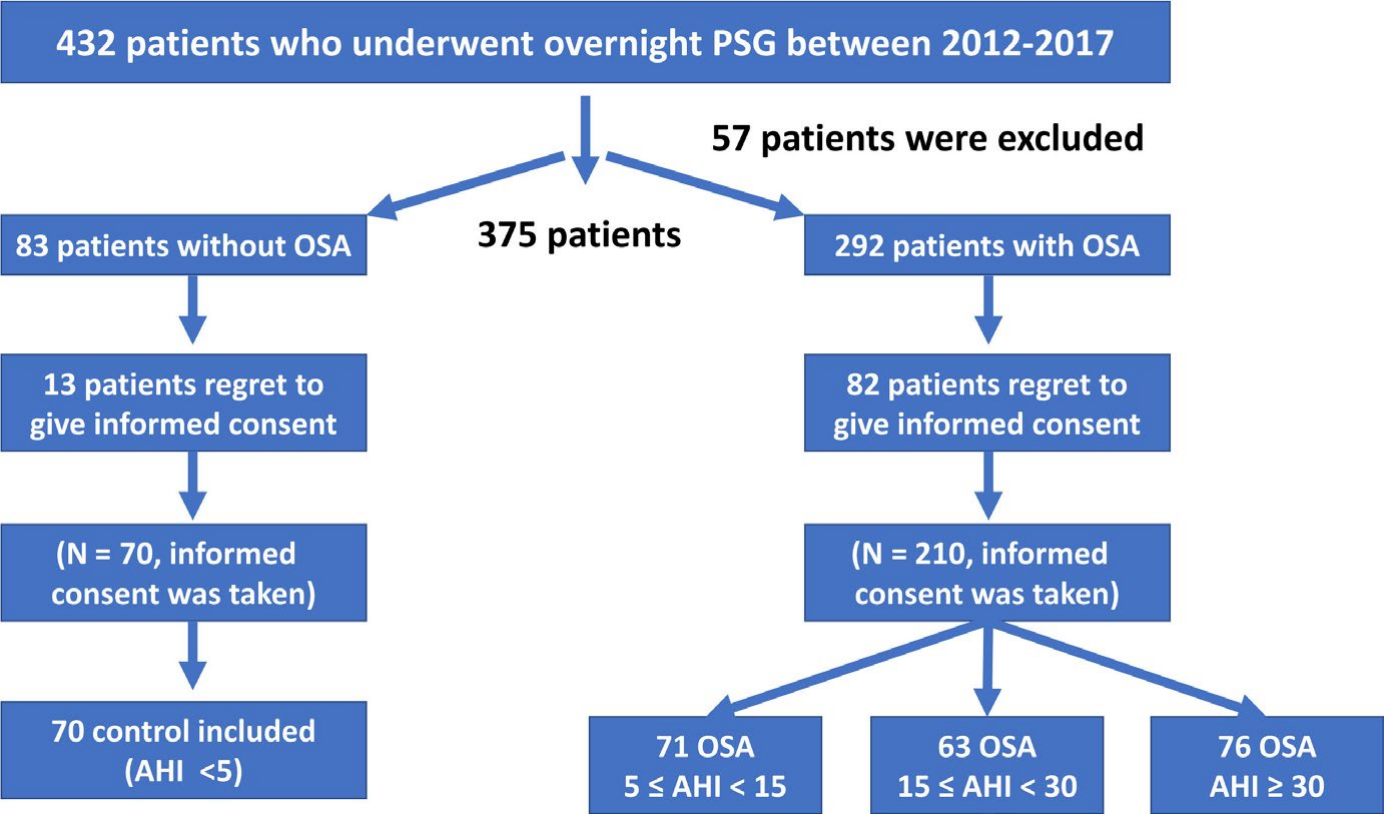
Flow chart of search strategy

We retrospectively reviewed the medical records of 432 patients who underwent overnight PSG between the years 2012 and 2017 in the sleep laboratory of the Pulmonary Medicine Department of Bezmialem University. Nocturnal snoring and excessive daytime symptoms were two main complaints, and all patients were asked questions from the Epworth sleepiness scale (ESS) (Johns, 1991), and patients with high scores (ESS ≥ 10) were accepted into the sleep study. Flow chart of search strategy was summarized in Figure 1. 210 consecutive patients who received the diagnosis of OSA were selected as patient group. Patient group were divided into following three subgroups: 71 with 5 ≤ AHI < 15, 63 with 15 ≤ AHI < 30, and 76 with AHI ≥ 30. Patients with normal PSG (70 patients; AHI < 5) formed the control group. After taking informed consent and identifying the groups, we reviewed the medical records of each patient. The day patients underwent PSG was assumed as the first day. Appropriate electrocardiographies (ECG) and echocardiographies which were performed as the shortest duration as sleep studies were accepted and evaluated. Patients with hypertension was diagnosed by a systolic blood pressure of 140 mm Hg or higher, or a diastolic blood pressure of 90 mm Hg or higher by at least three different measurements, or the use of antihypertensive medication. The diagnosis of diabetes mellitus was established by a fasting blood glucose of 7.0 mmol/L or higher, or the use of antidiabetic medication. Hyperlipidemia was defined as total cholesterol levels of 5.2 mmol/L or higher, or a history of statin use except in the last 3 months. Coronary artery disease was established when there was documentation of at least ≥ 70% of one major epicardial coronary vessel stenosis at the time of previous coronary angiography or had either a percutaneous coronary intervention (PCI), or postcoronary artery bypass graft (CABG). Patients smoking before hospitalization were accepted as smokers. The estimated glomerular filtration rate (eGFR) was calculated using the Chronic Kidney Disease Epidemiology Collaboration equation. Patients with any of the followings were excluded: atrial fibrillation, second or third degree atrioventricular blocks, previous pacemaker implantation, ECG without clearly analyzable QT segment, type I and III antiarrhythmic usage, pericarditis, valvular heart disease, cardiomyopathies, end‐stage hepatic and renal failure, craniofacial or respiratory disorders, and any form of sleep disorders other than OSA. The study was conducted in accordance with the Declaration of Helsinki, Good Clinical Practice and International Conference on Harmonization guidelines.

### 2.2 Electrocardiography

Standard 12‐lead ECG was recorded at 25 mm/s paper speed and 10 mm/mv amplitude in the supine position for each subject. All ECG recordings were scanned and transferred to the digital platform. The QT interval was measured from the beginning of the QRS complex to the end of T wave and QTc for the heart rate using the Bazett's Formula QTc = QT√RR interval. The QTd was defined as the difference between the maximum and minimum QT values, and the mean value of two consecutive cycles was calculated. Tp‐Te interval was defined as the interval from the peak of a T wave to the end of T wave. Measurements of Tp‐Te were performed from the precordial leads, lead V2 was selected for measuring. Tp‐Te/QT and Tp‐Te/QTc were calculated as the ratio of Tp‐Te to the corresponding QT and QTc interval in lead V2. Tp‐Te, Tp‐Te/QT, and Tp‐Te/QTc were calculated for each subject. ECG measurements of QT and Tp‐Te were performed by two cardiologists blinded to the patient data.

### 2.3 Echocardiography

Standard comprehensive transthoracic echocardiography was performed for each patient. Echocardiographic assessment was performed by using a VIVID 7 Dimension Cardiovascular Ultrasound System (Vingmed‐General Electric, Horten, Norway) with a 3.5 MHz transducer. Left ventricle diameters were measured using M‐mode imaging and left atrial diameter was evaluated in the four chamber apical view. Ejection fraction was calculated by using modified Simpson method. All echocardiographic examinations were performed by an experienced cardiologist.

### 2.4 Polysomography and sleep evaluation

All patients underwent overnight PSG (Comet As‐40, Grass Technology, Astro‐Med Inc., West Warwick, RI, USA) using international standards. The same device was used for all patients. Sleep studies were performed in Bezmialem University Sleep Laboratory.

The components of PSG in the sleep laboratory were continuous electroencephalographic (EEG) polygraphic recording using EEG leads, the use of right and left electro‐oculo‐graphic leads, chin electromyography for sleep staging, ECG, and arterial oxygen saturation by pulse oximetry. All sleep studies were interpreted by certified sleep physicians according to the manual of the American Academy of Sleep Medicine (AASM) for the Scoring of Sleep and Associated Events. Objective OSA severity was evaluated using the AHI, respiratory disturbance index (RDI), and minimal arterial oxygen saturation (MinSaO2). RDI is defined as the average number of episodes of apnea, hypopnea, and respiratory‐effort‐related arousals per hour. Apnea is identified when the airflow amplitude in the nasal cannula is < 10% of baseline and when no flow occurs on the airflow sensor. Hypopnea is identified when the amplitude of the airflow is reduced by 30% from the baseline, and the event is followed by 4% O_2_ desaturation. AHI is the combined average number of apneas and hypopneas that occur per hour of sleep. It is categorized into mild (5–15 events/hr), moderate (15–30 events/hr), and severe (>30 events/hr).

There are three common indications for respiratory sleep studies: (i) Diagnostic studies: to aid making a diagnosis, (ii) Intervention studies: to implement and titrate or confirm effectiveness of a new treatment, and (iii) Follow‐up studies: to follow the progress of a patient. Specifically the diagnosis, continuous positive airway pressure (CPAP) titration, and assessment of treatment results are the main indications for sleep studies in OSA.

### 2.5 Statistical analysis

Data analysis was performed by SPSS 17 (SPSS Inc., Chicago, IL, USA) package software. Quantitative variables are expressed as mean ± standard deviation and qualitative variables as numbers and percentages. After employing normality tests for understanding the distribution characteristics of the data, one‐way ANOVA with post hoc Tukey's test for the comparison between groups was used. Pearson correlation analysis was performed for correlation between RDI and repolarization parameters. An exploratory evaluation of additional cut‐points was performed using the receiver operating characteristics (ROC) curve analysis. Moreover, univariate and multivariate logistic regression analyses were done to determine significant independent predictors of OSA severity. A *p* value < .05 was considered statistically significant. All *p* values were two sided.

## 3 RESULTS

### 3.1 Comparison of baseline characteristics, echocardiographic, and electrocardiographic findings

The demographic characteristics of all patients are listed in Table 1. There was no significant difference between the groups in terms of gender, hypertension, hyperlipidemia, diabetes mellitus, coronary artery disease, and GFR. Patients with higher AHI tended to be older. Moreover smoking status and body mass index (BMI) were higher in patients with greater AHI. As the severity of AHI increased, lowest SPO2 decreased, while RDI increased. Echocardiographic findings showed patients with higher AHI had significantly elevated left atrial diameter, interventricular septal diameter (IVSD), and posterior wall diameter (PWD). Other parameters including ejection fraction, left ventricular end‐diastolic diameter (LVEDD), and pulmonary arterial pressure (PAP) were similar between four groups (Table 2).

**Table 1:** Patient characteristics by obstructive sleep apnea severity

	Control < 5 (Group 1)	AHI 5–14 (Group 2)	AHI 15–29 (Group 3)	AHI ≥ 30 (Group 4)	*p* Value
*N*	70	71	63	76	
Age	55.9 ± 5.3	55.2 ± 5.7	55.8 ± 5.0	59.0 ± 6.1	<.001
Male (%)	41 (58.6)	41 (58.6)	42 (60)	43 (61.4)	.984
BMI category (kg/m^2^) (%)					
<25 (%)	3 (4.2)	6 (8.5)	3 (4.2)	1 (1.4)	.001
25–30 (%)	27 (38.5)	21 (30)	29 (41.4)	14 (20)	.029
≥30 (%)	40 (57.1)	43 (61.4)	38 (68.5)	55 (78.5)	<.001
Hypertension (%)	51 (72.8)	52 (74.2)	55 (78.5)	55 (78.5)	.804
Hyperlipidemia (%)	6 (8.57)	7 (10)	8 (11.4)	4 (5.7)	.677
Coronary artery disease (%)	41 (58.5)	47 (67.1)	48 (68.5)	47 (67.1)	.589
DM (%)	23 (32.8)	24 (34.2)	25 (35.7)	24 (34.2)	.989
Smoking (%)	15 (21.4)	27 (38.5)	27 (38.5)	36 (51.4)	.003
GFR (ml/min/1.73 m^2^)	65.9 ± 29.1	66.0 ± 9.5	66.2 ± 10.3	65.7 ± 11.0	.953
RDI	10.1 ± 2.7	12.3 ± 2.5	21.8 ± 3.5	31.0 ± 4.4	<.001
Mean SpO2 (%)	93.9 ± 1.9	93.9 ± 1.9	92.7 ± 3.3	92.4 ± 4.1	.004
Lowest SpO2 (%)	83.9 ± 2.6	83.9 ± 2.6	83.9 ± 2.5	80.5 ± 5.9	<.001

Note

Expressed as mean ± *SD* and *N* (%) for continuous and categorical variables, respectively.

Abbreviations: AHI, apnea–hypopnea index, BMI, body mass index; DM, diabetes mellitus; GFR, glomerular filtration rate; RDI, respiratory disturbance index; SpO2, arterial oxygen saturation.

**Table 2:** Baseline echocardiographic characteristics

	Control < 5 (Group 1)	AHI 5–14 (Group 2)	AHI 15–29 (Group 3)	AHI ≥ 30 (Group 4)	*p* Value
LVEDD (mm)	50.7 ± 3.5	50.5 ± 3.5	50.6 ± 3.5	50.6 ± 3.5	.987
Left atrial diameter (mm)	39.7 ± 2.4	39.8 ± 2.4	39.8 ± 2.4	41.0 ± 3.1	.009
IVSD (mm)	10.3 ± 2.0	10.3 ± 2.0	11.2 ± 1.7	11.9 ± 0.8	<.001
PWD (mm)	10.5 ± 2.0	10.6 ± 1.9	11.5 ± 1.5	11.9 ± 0.8	<.001
LVEF (mean %)	54.2 ± 10.2	54.5 ± 11.6	54.6 ± 11.1	52.4 ± 12.3	.645
PAP (mmhg)	35.6 ± 12.4	37.4 ± 13.6	39.5 ± 13.5	36.8 ± 14.5	.382

Note

Expressed as mean ± *SD*.

Abbreviations: AHI, apnea–hypopnea index; IVSD, interventricular septal diameter; LVEDD, left ventricular end‐diastolic diameter; LVEF, left ventricular ejection fraction; PAP, pulmonary artery systolic pressure; PWD, posterior wall diameter.

Compared to control group, repolarization parameters were significantly higher in OSA groups. These parameters were significantly different between four groups, respectively (*p* < .001). There was a significant trend toward higher Tp‐Te, Tp‐Te/QT, and Tp‐Te/QTc across higher AHI categories (Table 3).

**Table 3:** Electrocardiographic findings of the study population

	Control < 5 (Group 1)	AHI 5–14 (Group 2)	AHI 15–29 (Group 3)	AHI ≥ 30 (Group 4)	*p* Value
Heart rate (beats/min)	73 ± 8	76 ± 10	75 ± 9	72 ± 12	.680
QT (ms)	386 ± 24	388 ± 32	397 ± 26	406 ± 28	<.001
QTc	426 ± 21	437 ± 28	444 ± 23	445 ± 26	<.001
QTd	37.4 ± 5.5	38.1 ± 5.5	43.6 ± 5.8	48.5 ± 7.7	<.001
Tp‐Te (ms)	68.3 ± 6.8	71.8 ± 6.3	79.1 ± 5.5	85.1 ± 6.4	<.001
Tp‐Te/QT	167.5 ± 12.7	181.7 ± 13.0	202.2 ± 10.0	219.4 ± 13.5	<.001
Tp‐Te/QTc	151.1 ± 16.6	167.6 ± 16.6	193.7 ± 14.4	225.5 ± 17.0	<.001

Note

Expressed as mean ± *SD*.

Abbreviations: AHI, apnea–hypopnea index; QTc, corrected QT interval; QTd, QT interval dispersio; Tp‐Te, T peak and T end interval.

The correlation between repolarization heterogeneity parameters (Tp‐Te/QTc) and standard QT measurements including QTc (*r* = 0.554; *p* < .001) and QTd (*r* = 0.529; *p* < .001) were also recorded (Table 3). As it was expected, standard QT parameters correlated with the grade of OSA severity.

**Figure 2:**
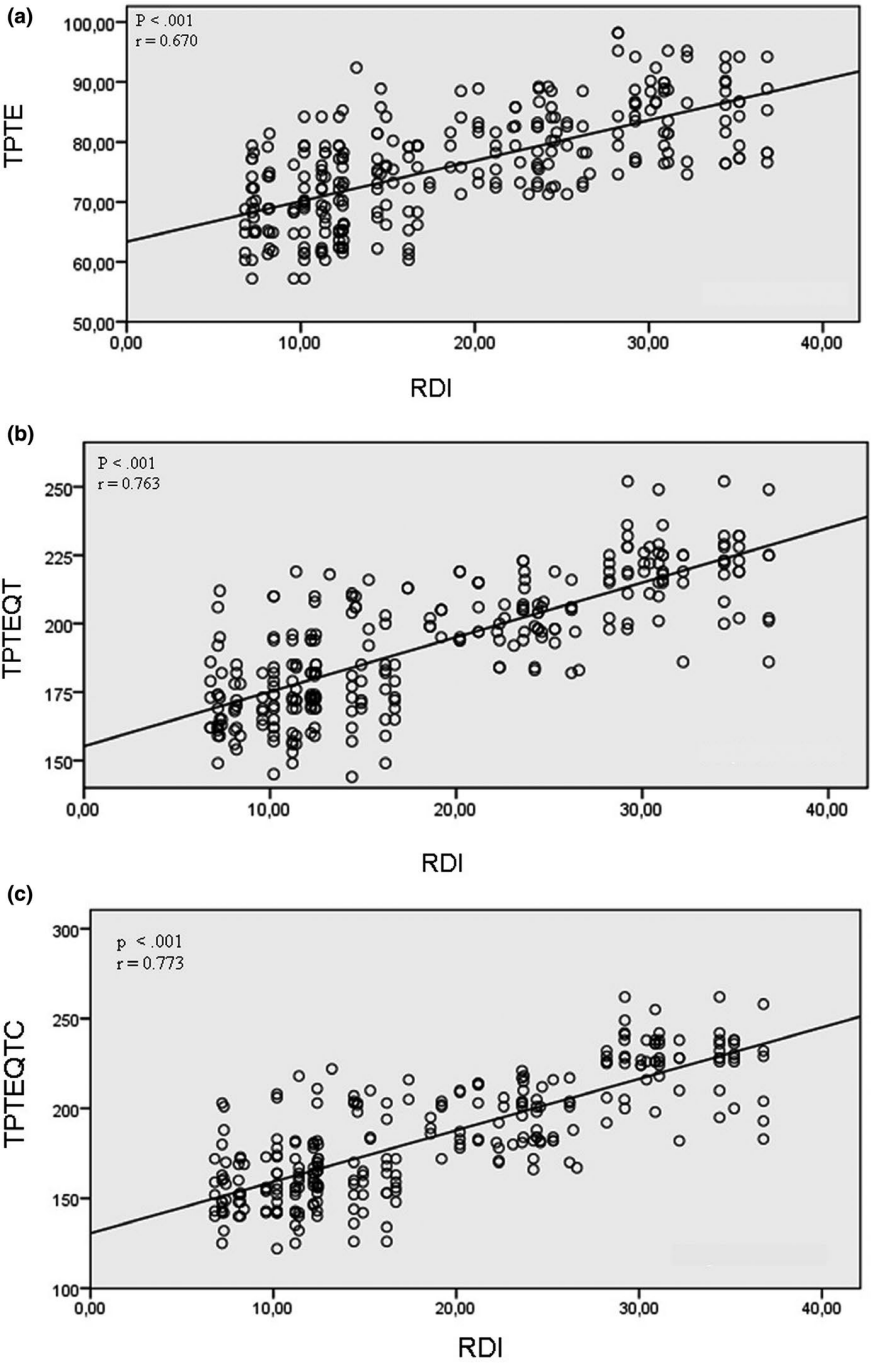
(a) Correlation between Tp‐Te interval and respiratory disturbance index. (b) Correlation between Tp‐Te/QT ratio and respiratory disturbance index. (c) Correlation between Tp‐Te/QTc ratio and respiratory disturbance index

Additionally there was a significant positive correlation among RDI and Tp‐Te (*r* = 0.670; *p* < .001), Tp‐Te/QT (*r* = 0.763; *p* < .001), and Tp‐Te/QTc (*r* = 0.773; *p* < .001) (Figure 2–1, 2–2, 2–3).

### 3.2 Predicting OSA severity

**Figure 3:**
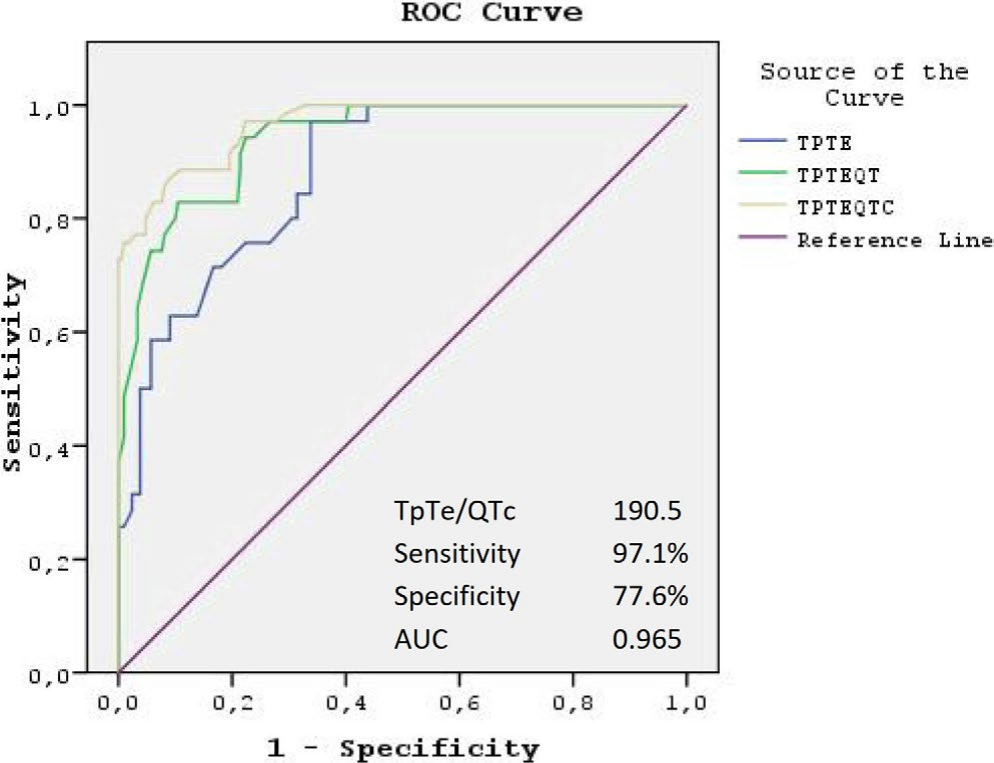
Receiver operating characteristic (ROC) curve comparison of Tp‐Te interval, Tp‐Te/QT, and Tp‐Te/QTc ratios for prediction of OSA severity

To identify the independent predictors of OSA severity, a multivariate logistic regression analyses was performed using the variables that showed marginal association with OSA severity in the univariate analyses. These variables are shown in Table 4. Tp‐Te (OR 1.629, 95% CI 1.393–1.906, *p* < .001), Tp‐Te/QTc (OR 1.333, 95% CI 1.247–1.424, *p* < .001), and smoking (OR 5.771, 95% CI 1.025–32.479, *p* = .047) were found to be significant independent predictors of severity of AHI in a multivariate analysis. We performed ROC analysis and demonstrated that Tp‐Te > 77.2 ms predicted severity of OSA with a sensitivity of 0.843 and a specificity of 0.680 (AUC 0.880, 95% CI 0.839–0.920; *p* < .001). Tp‐Te/QTc > 190.5 had higher sensitivity (0.971) and specificity (0.776) (AUC 0.965, 95% CI 0.945–0.985; *p* < .001) respectively (Figure 3).

**Table 4:** Univariate and multivariate regression analyses of predictors of disease severity

Variables	Univariate analysis		Multivariate analysis	
	Odds ratio (95% CI)	*p*	Odds ratio (95% CI)	*p*
Age	0.811 (0.932–1.056)	.661		
Gender	0.126 (0.573–2.216)	.730		
BMI (kg/m^2^)	1.317 (1.146–1.513)	<.001	1.373 (0.987–1.911)	.060
Smoking	3.882 (1.855–8.126)	<.001	5.771 (1.025–32.479)	.047
Hypertension	1.366 (0.628–2.970)	.431		
Tp‐Te (ms)	1.537 (1.401–1.686)	<.001	1.629 (1.393–1.906)	<.001
Tp‐Te/QTc	1.309 (1.241–1.380)	<.001	1.333 (1.247–1.424)	<.001
PAP (mmHg)	1.007 (0.981–1.033)	.606		
EF (mean %)	0.987 (0.959–1.016)	.375		
GFR (ml/min/1.73 m^2^)	0.999 (0.980–1.019)	.940		

Abbreviations: BMI, body mass index; EF, ejection fraction; GFR, glomerular filtration rate; PAP, pulmonary artery systolic pressure; QTc, corrected QT interval; Tp‐Te, T peak and T end interval.

## 4 DISCUSSION

Tp‐Te, Tp‐Te/QT, and Tp‐Te/QTc were found to be prolonged in patients with OSA. Severity of OSA as determined by AHI was associated with a higher likelihood of having abnormally increased repolarization parameters. These associations were independent of factors such as age, sex, BMI, and smoking that were potential confounders.

In general, patients with higher AHI are older and have male predominance and higher blood pressure. In concordance with previous studies, patients with severe OSA were older in this study. However, there was no difference between the groups in terms of gender, hypertension, and coronary artery disease. Echocardiography revealed severe left ventricular hypertrophy in patients with higher AHI. According to this finding, hypertensive patients could be underdiagnosed. Moreover, patients who were excluded from the study due to severe comorbidities were predominantly male, which may be a logical explanation for equal gender distribution in all groups.

The QT interval represents ventricular depolarization and repolarization phases. It is considered to be a marker for ventricular electrical instability; hence, it includes vulnerable period for ventricular malignant arrhythmias via reentry mechanisms (Moss, 2006). Increased vagal activity causes significant QT prolongation during apneic periods in OSA. On the other hand, increased sympathetic tone causes abrupt QT shortening during postapneic periods (Leung, 2009). QT and QTc were found to be prolonged in patients with higher AHI groups in this study. However, the study was not designed to investigate QT and QTc alterations in response to apneic and postapneic episodes.

Hypoxia, hypercapnia, and acidemia also play a role in QT prolongation (Roche et al., 2003). Lowest SpO2 and highest RDI were recorded in severe OSA group. Additionally there was a significant correlation between RDI and Tp‐Te, Tp‐Te/QT, and Tp‐Te/QTc.

QT‐corrected interval dispersion (QTcd) is the difference between the maximum and the minimum QT intervals on ECG and shows both electrical instability and inhomogeneity in repolarization (Malik & Batchvarov, 2000). The Rotterdam Study and large prospective studies (Bruyne et al., 1998; Perkiomaki et al., 1995) demonstrated an increased QTcd was a strong and independent risk factor for cardiac mortality and malignant ventricular arrhythmias. Further, QTcd was shown to be increased in patients with OSA (Dursunoglu et al., 2005). CPAP therapy improved the inhomogeneity of repolarization via a significant decrease in QTcd even after six months of CPAP usage (Dursunoglu & Dursunoglu, 2007). Nakamura et al. reported QTcd was prolonged in patients with OSA during sleep (Nakamura et al., 2004). However, Barta et al. failed to show QTcd increase during sleep and they did not find an increased risk of ventricular arrhythmias (Barta et al., 2010). Patients with higher AHI and severe comorbidities in the first study may be substantial reasons for these conflicting results.

Tp‐Te/QT predicts more accurately the ventricular arrhythmias than Tp‐Te, because it is not influenced from variations in heart rate and body weight. Previous studies demonstrated that increased Tp‐Te was associated with mortality in primary percutaneous coronary intervention for myocardial infarction, hypertrophic cardiomyopathy, Brugada syndrome, and long QT syndrome (Haarmark et al., 2009; Shimizu et al., 2002). Prolongation of Tp‐Te has also been reported in OSA (Kilicaslan et al., 2012). Sökmen et al. postulated in his study that Tp‐Te, Tp‐Te/QT, and Tp‐Te/QTc increased during apnea period and decreased during the postapnea hyperventilation period (Sökmen et al., 2018). On the other hand, QTc persisted to be prolonged instead of shortening during postapnea hyperventilation period. Novel parameters including Tp‐Te, Tp‐Te/QT, and Tp‐Te/QTc may be more sensitive to the changes in autonomic nervous system and hypoxia than QTc. Sökmen et al. did not evaluate the association between AHI and ventricular repolarization parameters in their study. Results should be interpreted based on AHI severity in OSA studies.

Strength of our study includes the analysis of a relatively large number of OSA patients. It gives an opportunity to stratify patients according to severity of AHI. As a result, the larger sample size allowed multivariate adjustment of age, BMI, and smoking history, both of which may have significant effects on inter‐individual variations in Tp‐Te and QT interval. This study could be regarded as comprehensive and confirmatory study of all previous small studies.

Cardiac arrhythmias (Shamsuzzaman et al., 2003) and SCD (Pearce & Saunders, 2003) are common in OSA, and these arrhythmias correlate with degree of oxygen desaturation and severity of AHI (Ryan et al., 2005). Risk assessment of life‐threatening arrhythmias and SCD is of paramount importance especially among individuals without established cardiovascular disease. We found increased ventricular repolarization heterogeneity in OSA. It is associated with a higher likelihood of inducing ventricular arrhythmias. Tp‐Te, Tp‐Te/QT, and Tp‐Te/QTc may play a role in risk assessment of life‐threatening arrhythmias and SCD.

This study has several limitations. Although sample size is large enough, the study is single‐centered and has a retrospective design. A prospective multicenter study may allow better assessment of correlation between AHI and Tp‐Te, Tp‐Te/QT, and Tp‐Te/QTc in OSA. Further studies were needed to build a deeper look with repolarization parameters to predict the prognostic role in arrhythmias.

## 5 CONCLUSIONS

There is a significant correlation between AHI and Tp‐Te interval, Tp‐Te/QT, and Tp‐Te/QTc ratios in patients with OSA.

## CONFLICT OF INTEREST

The authors declare that they have no conflict of interest. The authors alone are responsible for the content and writing of the article. The authors have had full control of all primary data and they agree to allow the journal to review their data if requested.

## AUTHOR CONTRIBUTIONS

*Conceived and designed the analysis:* EK, HBK

*Collected the data:* HBK

*Contributed to data or analysis tools:* EK, HBK

*Performed the analysis:* EK

*Wrote this paper:* EK

## ETHICAL APPROVAL

All procedures performed in studies involving human participants were in accordance with the ethical standards of the institutional and/or national research committee and with the 1964 Helsinki declaration and its later amendments or comparable ethical standards.

## Data Availability

The data that support the findings of this study are available from the corresponding author upon reasonable request.
